# Supplementary material for: Bidirectional Mediation Effects between Intratumoral Microbiome and Host DNA Methylation Changes Contribute to Stomach Adenocarcinoma
Source: Microbiol Spectr. 2023 Jun 1;11(4):e00904-23. doi: 10.1128/spectrum.00904-23 (PMC10434028; doi:10.1128/spectrum.00904-23)
Supplement: Supplemental file 1 — Supplemental materials and methods and Fig. S1 to S5. Download spectrum.00904-23-s0001.docx, DOCX file, 7.5 MB [file spectrum.00904-23-s0001.docx]

**Supplement materials and methods**

**Bi-mediation analysis**

Survival mediation analyses : 1) Fit aalen (surv (OS time, OS) ~ X) to obtain the TE. 2) Fit aalen (surv (OS time, OS) ~ X+M) to obtain the DE. The total effect minus the direct effect/total effect is the ratio of X's influence on Y through M.

Casual mediation analyses : 1) Explore the relationship between X on M and X and M on Y respectively. 2) perform mediation analysis to evaluate whether M is possible mechanisms underlying X on Y (mediation package). The effects of age and sex were corrected in each model.


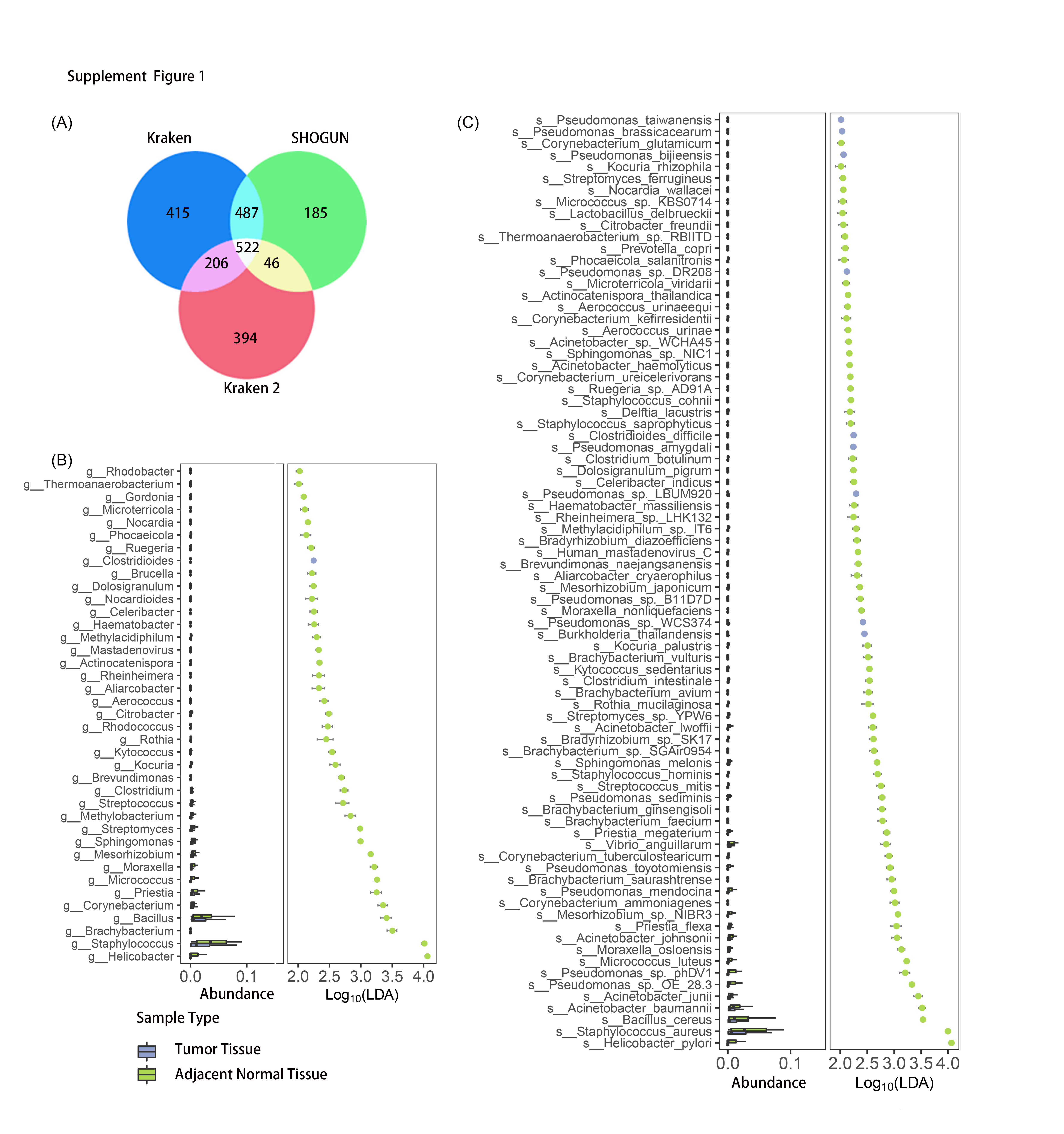


**Supplementary Figure 1. Differential analysis of tumor microbiota. A,** The tumor-associated genera screened by kraken (top left), SHOGUN (top right) and kraken2 (bottom). **B,C,** Differences in the abundance of genera (b) and species bacteria (c) between tumor and adjacent normal tissues.


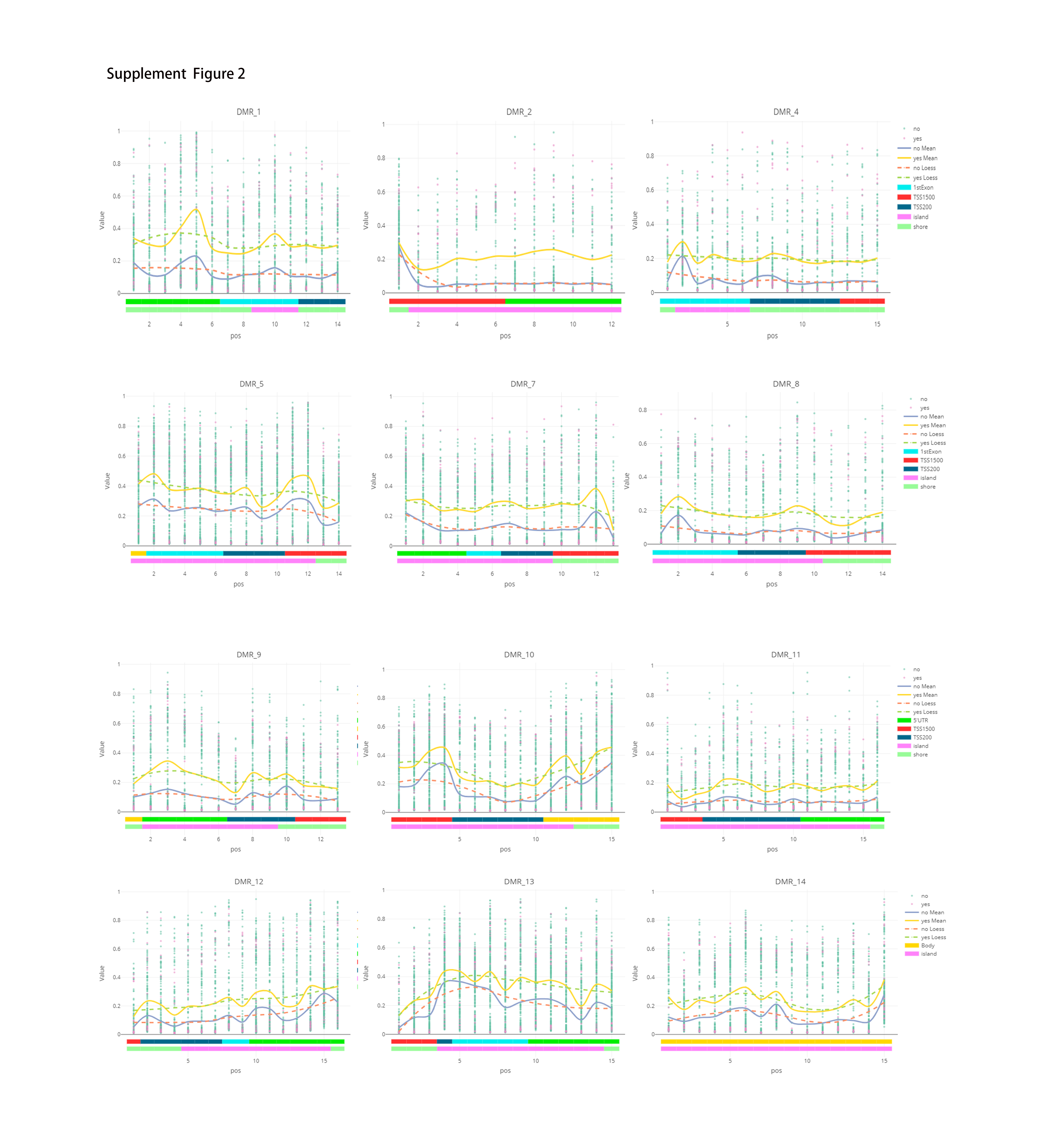


**Supplementary Figure 2. The CpGs information involved in the DMRs (metastasis).**


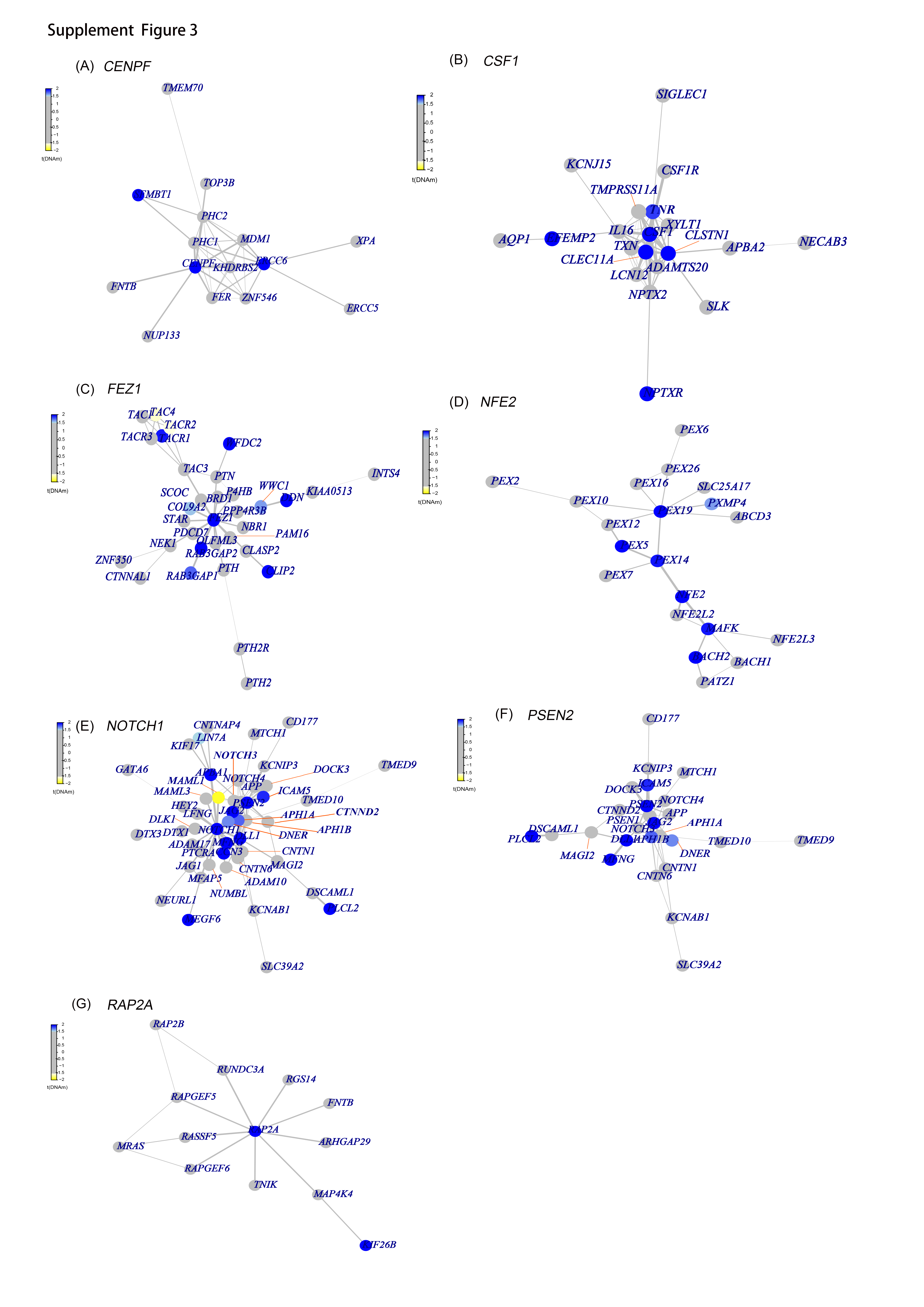


**Supplementary Figure 3. Interaction hotspot with *CENPF, CSF1, FEZ1, NFE2, NOTCH1, PSEN2* and *RAP2A* as the hub gene, separately**. The color of the node was defined according to the T value of differential methylation. The color below -1.5 was a gradual change from yellow to white, the color above 1.5 was a gradual change from light blue to blue, and the middle was gray.


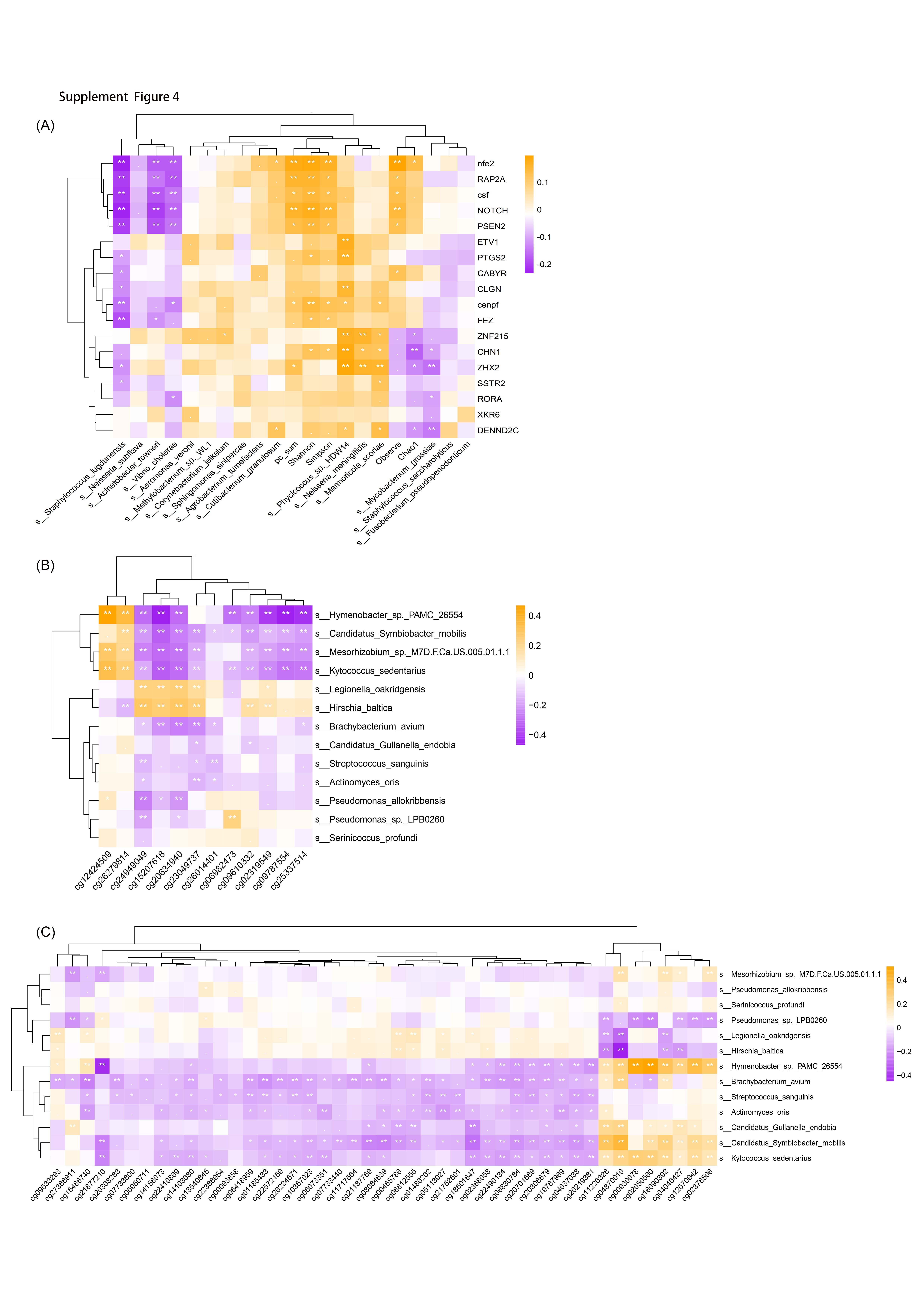


**Supplementary Figure 4. The relationship between the microbial characteristics and methylated features. A,** Heatmap display the relationship between the microbial characteristics, diversity indexes and DMRs, interaction hotspots related to the distant metastasis of cancer cells. **B, C**, The heatmap showed the correlation measured by Spearman rank correlation coefficient between 13 microbial characteristics and 12 DMPs (b) and 45 DMRs (c) related to prognosis of STAD patients. Yellow represents positive correlation and purple represents negative correlation. *: *p* <= 0.05, **: *p* <= 0.01, ***: *p* <= 0.001, ****.

**Supplementary Figure 5**

**
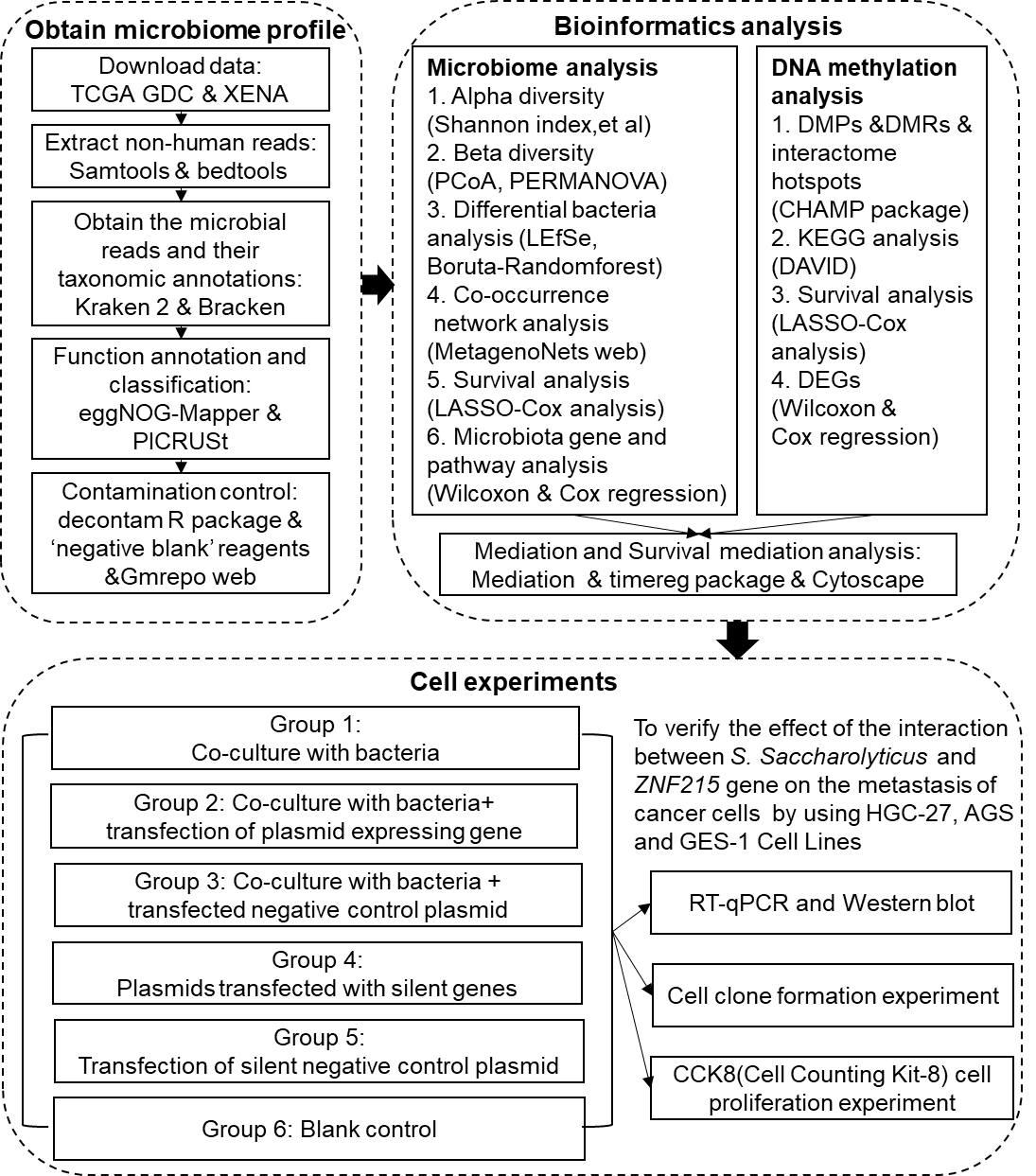
**

**Supplement Figure 5. The flow chart of manuscript.**
